# The relapsing fever spirochete *Borrelia miyamotoi* is cultivable in a modified Kelly-Pettenkofer medium, and is resistant to human complement

**DOI:** 10.1186/1756-3305-7-418

**Published:** 2014-09-04

**Authors:** Alex Wagemakers, Anneke Oei, Michelle M Fikrig, Willem R Miellet, Joppe W Hovius

**Affiliations:** Center for Experimental and Molecular Medicine, Academic Medical Center, Meibergdreef 9, 1105 AZ Amsterdam, The Netherlands; Department of Medical Microbiology, Academic Medical Center, Amsterdam, The Netherlands; Department of Internal Medicine, Division of Infectious Diseases, Academic Medical Center, Amsterdam, The Netherlands; Amsterdam Multidisciplinary Lyme Center, Academic Medical Center, Amsterdam, The Netherlands

**Keywords:** Culture, MKP, *Borrelia miyamotoi*, Relapsing fever, Complement resistance, *Borrelia anserina*

## Abstract

**Background:**

*Borrelia miyamotoi* is a relapsing fever spirochete found in *Ixodes* ticks in North America, Europe, and Asia, and has recently been found to be invasive in humans. Cultivation of this spirochete has not yet been described, but is important for patient diagnostics and scientific purposes. Host specificity of *Borrelia* species is dependent on resistance to host complement (serum resistance), and since *B. miyamotoi* has been identified as a human pathogen we were interested whether *B. miyamotoi* is resistant to human complement.

**Methods:**

We inoculated *B. miyamotoi* strains LB-2001 and HT31 in modified-Kelly-Pettenkofer medium with 10% fetal calf serum (MKP-F), and used standard non-laborious *Borrelia* culture methods to culture the spirochetes. Next, we assessed serum sensitivity by a direct killing assay and a growth inhibition assay.

**Results:**

We were able to passage *B. miyamotoi* over 10 times using a standard culture method in MKP-F medium, and found *B. miyamotoi* to be resistant to human complement. In contrast to *B. miyamotoi*, *Borrelia anserina* - a relapsing fever spirochete unrelated to human infection- was serum sensitive.

**Conclusions:**

Using a variation on MKP medium we were able to culture *B. miyamotoi*, opening the door to *in vitro* research into this spirochete. In addition, we describe that *B. miyamotoi* is resistant to human complement, which might play an important role in pathogenesis. We have also found *B. anserina* to be sensitive to human complement, which might explain why it is not related to human infection. Summarizing, we describe a novel culture method for *B. miyamotoi* and show it is resistant to human complement.

## Background

*Borrelia miyamotoi* is a relapsing fever spirochete first discovered in *Ixodes persulcatus* ticks in Hokkaido, Japan [1], which over the years has been found across North- America in *Ixodes pacificus* and *Ixodes scapularis* ticks [2, 3] and Europe [4], where it has been identified in *Ixodes ricinus* ticks. The first human cases of *B. miyamotoi* infection have only recently been identified in Russia [5], and together with studies performed in the U.S.A. [6, 7], demonstrated a clinical picture of a febrile viral-like illness several weeks after a tick-bite. In two patients with severe immunodeficiency, *B. miyamotoi* infection was found to cause a meningoencephalitis [8, 9]. Detection of *B. miyamotoi* infection in patients and ticks is mostly performed by PCR for the 16S rRNA gene, flagellin or the GLPQ gene. Serology is currently based on detection of anti-GLPQ antibodies. While Asian *B. miyamotoi* strains have been isolated using BSK-II medium [1], there are no reports describing its consistent *in vitro* propagation. In addition, established methods for propagating the North-American strain LB-2001 rely on intraperitoneal inoculation in SCID mice and this strain in particular is considered to be uncultivable [10]. For both diagnostic and scientific purposes a practical and non-laborious culture method for this relatively unknown spirochete should be established. This method should ideally not differ much from the culture methods employed for other relapsing fever and *B. burgdorferi sensu lato* spirochetes. We have tested multiple culture media modifications and here we describe one in particular that allowed us to culture *B. miyamotoi* in a medium and method that also readily propagates various other *Borrelia* spirochetes. Serum sensitivity differs greatly amongst relapsing fever as well as *B. burgdorferi* sensu lato species, and is thought to be important in its ecology, capacity to invade different hosts and human pathogenesis [11]. Since we were now able to culture *B. miyamotoi*, we explored the susceptibility of *B. miyamotoi* to human complement (serum sensitivity).

## Methods

### *Borrelia*strains

*B. miyamotoi* strain LB-2001 was derived from *I. scapularis* ticks in the U.S.A. [2] and had been propagated through intraperitoneal inoculation of SCID mice approximately ten times since its isolation from a tick. *B. miyamotoi*-infected plasma from a SCID mouse was kindly provided by Durland Fish and Linda Bockenstedt, Yale University. *B. miyamotoi* strain HT31 was isolated in BSK-II medium from an *I. persulcatus* tick in Japan [1] and a low-passage (less than 5) isolate was provided by Barbara Johnson, CDC through Volker Fingerle, German National Reference Centre for *Borrelia*. Low-passage (less than 5 passages since their isolation) *B. hermsii* HS1 [12], *B. anserina* Ni-NL [13, 14] and *B. garinii* strain A87S [15] were cultured from −80°C glycerolpeptone stocks. High-passage (more than 20) reference strain *B. afzelii* PKo [16, 17] was inoculated in a C3H mouse through intradermal syringe injection and a low-passage (less than 5) bladder isolate was cultured from −80°C glycerolpeptone stocks for *in vitro* experiments.

### Description of culture medium and culture conditions

The culture medium we used for culturing *B. miyamotoi* is a variation on modified Kelly-Pettenkofer Medium, designated MKP-F. One liter of medium is prepared as follows: First, 162.8 ml Milli-Q water, 65.1 ml 10× CMRL 1066 without glutamine (Life technologies, Carlsbad, CA, U.S.A.), 44.8 ml heat-inactivated rabbit serum (Biotrading, Mijdrecht, The Netherlands), 3.9 g of HEPES (Sigma-Aldrich, St. Louis, MO, U.S.A.), 3.3 g of glucose (Sigma-Aldrich), 2.0 g of Neopeptone (BD biosciences, Franklin lakes, NJ, U.S.A.), 1.4 g of sodium bicarbonate (Sigma-Aldrich), 523 mg of Sodium pyruvate (Merck Millipore, Billerica, MA, U.S.A.), 458 mg of sodium citrate (Sigma-Aldrich), and 261 mg of N-acetyl-glucosamine (Sigma-Aldrich) were prepared, set to pH 7.6 by adding 10 N NaOH (Merck Millipore), and filtered using a 0.2 μm filter. Next, 500 ml of 65.57 g/L bovine serum albumin (Sigma-Aldrich) in Milli-Q water (filtered and pH set to 7.6) was added. Finally, 127.3 ml of autoclaved 7% gelatin (Oxoid, Thermo Scientific, Waltham, MA, U.S.A.) set to pH 7.7 and 100 ml heat-inactivated fetal calf serum (BioWhittaker- Lonza, Basel, Switzerland) were added. Seven milliliter aliquots in nine milliliter sterile glass tubes (VSM, Andeville, France) were stored at −20°C until use. A total of 500 μl of plasma from an LB-2001 infected SCID mouse or from medium containing *B. miyamotoi* HT31 was added to room-temperature MKP-F medium and capped tubes were incubated in a 33°C incubator (Memmert, Schwabach, Germany), creating a microaerophilic environment. After 6–8 days cultures had reached approximately 1–2 × 10^7^/ml) and were subsequently passaged at 1:5 or 1:10 dilution for P2, 1:25 for P3 and 1:100 for all subsequent passages, or aliquotted and stored at −80°C in 4% glycerolpeptone. Spirochetes were enumerated directly as described previously [17], using dark-field microscopy on 5 μl samples by counting at least 5 fields at a 250x magnification. A total of 350 μl of cerebrospinal fluid (CSF) - that had been stored at −80°C for two years - from a previously described patient [9] was cultured in MKP-F and checked for the presence of viable spirochetes for 6 weeks, using dark-field microscopy.

### Serum sensitivity

All strains were cultured at 33°C using the above mentioned culture medium until they reached a concentration of 1-2×10^7^/ml, counted as described before [18]. For normal human serum (NHS) we pooled serum samples from 4 healthy individuals (stored in −80°C) in equal ratios, and heat-inactivated serum (HIS) was generated by incubating NHS at 56°C for 45 minutes. Serum samples were checked for the absence of *Borrelia burgdorferi* s.l. antibodies using a C6 EIA (Immunetics, Boston, MA, U.S.A.) and all were negative. In a 96-well V-shaped cell culture plate (Greiner bio-one, Kremsmünster, Austria) 25 μl of the spirochete culture and 25 μl of NHS or HIS were mixed and the plate was sealed and incubated at 37°C. After one and three hours, wells were resuspended and 5 μl of the samples were analyzed under dark-field microscopy. Samples were blinded and 100 spirochetes per sample were designated as either motile or immotile, as described previously [19]. Another method to assess serum sensitivity was performed using a pH indicator, based on previous studies in other *Borrelia* species [19–21]. In short, 5x10^6^ mid-log phase (1-2×10^7^/ml) *B. miyamotoi* LB-2001, *B. miyamotoi* HT31, *B. garinii* A87S and *B. anserina* spirochetes were washed in PBS, resuspended in 50 μl MKP-F medium containing a final phenol red concentration of 240 μg/ml, rifampicine (50 μg/ml) and phosphomycin (100 μg/ml). Samples were mixed with 50 μl pooled NHS or 50 μl HIS and cultured in sealed microtiter plates at 33°C for multiple days during which absorbance was measured daily at 562/630 nm using an ELISA plate reader (BioTek instruments inc., Winooski, VT, U.S.A.).

### Statistical analysis

A Kruskal-Wallis test was performed to identify a difference in motility between different *Borrelia* strains and conditions. The significance of the difference between two conditions (normal human serum versus heat-inactivated serum) for each *Borrelia* genospecies was analyzed using a two-tailed Mann–Whitney test. Optical densometry curves were compared using a repeated measures ANOVA. All analyses were performed using Prism 5.0 software (GraphPad Software, San Diego, CA) and p < 0.05 was considered significant.

## Results

### Culturing *B. miyamotoi*

Using a variation on Modified Kelly-Pettenkofer Medium, containing 10% FCS and designated MKP-F (Table 1), we managed to culture *B. miyamotoi* LB-2001 and *B. miyamotoi* strain HT31 in a similar fashion as we culture *B. burgdorferi* sensu lato in our laboratory. Using 7 ml of medium in 9 ml glass tubes in a 33°C incubator, we were able to consistently grow *B. miyamotoi* to a concentration of 1–2 × 10^7^/ml for 10 passages (Table 2), as well as culture *B. miyamotoi* from glycerolpeptone stocks stored at −80°C. In addition, there was morphologically no difference in spirochete viability and motility throughout passages as assessed by dark-field microscopy. However, a frozen CSF sample from a patient with *B. miyamotoi* meningoencephalitis remained negative after 6 weeks of cultivation.

**Table 1:** **Comparison of medium ingredients between MKP-F and the MKP medium it is based upon**

MKP-F	Per liter	MKP	Per liter
MilliQ	662.8 ml	MilliQ	670 ml
7% gelatine	127.3 ml	7% gelatine	149 ml
FCS	100 ml	-	
10× CMRL	65.1 ml	10x CMRL	74.5 ml
Rabbit serum	44.8 ml	Rabbit serum	53.6 ml
BSA	32.8 g	BSA	52.2 ml
HEPES	3.9 g	HEPES	4.5 g
Glucose	3.3 g	Glucose	2.2 g
Neopeptone	2.0 g	Neopeptone	3.7 g
Sodium bicarbonate	1.4 g	Sodium bicarbonate	1.5 g
Sodium citrate	0.5 g	Sodium citrate	0.5 g
Sodium pyruvate	0.5 g	Sodium pyruvate	0.6 g
N-acetyl glucosamine	0.3 g	N-acetyl glucosamine	0.3 g

**Table 2:** **Peak densities and motility in serial passages of** ***B. miyamotoi*** **in MKP-F medium**

Passage	Peak density ^#^		Motility*	
	LB-2001	HT31	LB-2001	HT31
**1**	12.5^1^	1.9^1^	90^1^	100^1^
**2**	15.2 (2.3)^2^	14.2 (4.2)^3^	100 (0)^2^	96.7 (3.3)^3^
**3**	21.6 (0.9)^2^	14.8 (4.7)^3^	100 (0)^2^	100 (0)^3^
**4**	14.4 (0.6)^2^	22.3 (5.5)^3^	100 (0)^2^	100 (0)^3^
**5**	18.1 (4.4)^2^	15.8 (4.9)^3^	100 (0)^2^	100 (0)^3^
**6**	11.6 (0.3)^2^	20.2 (4.2)^3^	100 (0)^2^	96.7 (3.3)^3^
**7**	15.0 (1.3)^2^	21.9 (4.5)^3^	100 (0)^2^	100 (0)^3^
**8**	20.0 (2.5)^2^	19.6 (6.0)^3^	100 (0)^2^	100 (0)^3^
**9**	19.1 (9.1)^2^	24.0 (7.8)^3^	100 (0)^2^	100 (0)^3^
**10**	16.9 (5.6)^2^	17.7 (4.5)^3^	100 (0)^2^	100 (0)^3^

*B. miyamotoi* strains LB-2001 and HT31 were successfully passaged to P10 multiple times. ^#^Mean (±SEM) ×10^6^ spirochetes/ml as determined by dark-field microscopy. *Motility is depicted as the mean percentage of motile spirochetes (±SEM). The number of individual cultures is depicted in superscript.

### Serum sensitivity

**Figure 1:**
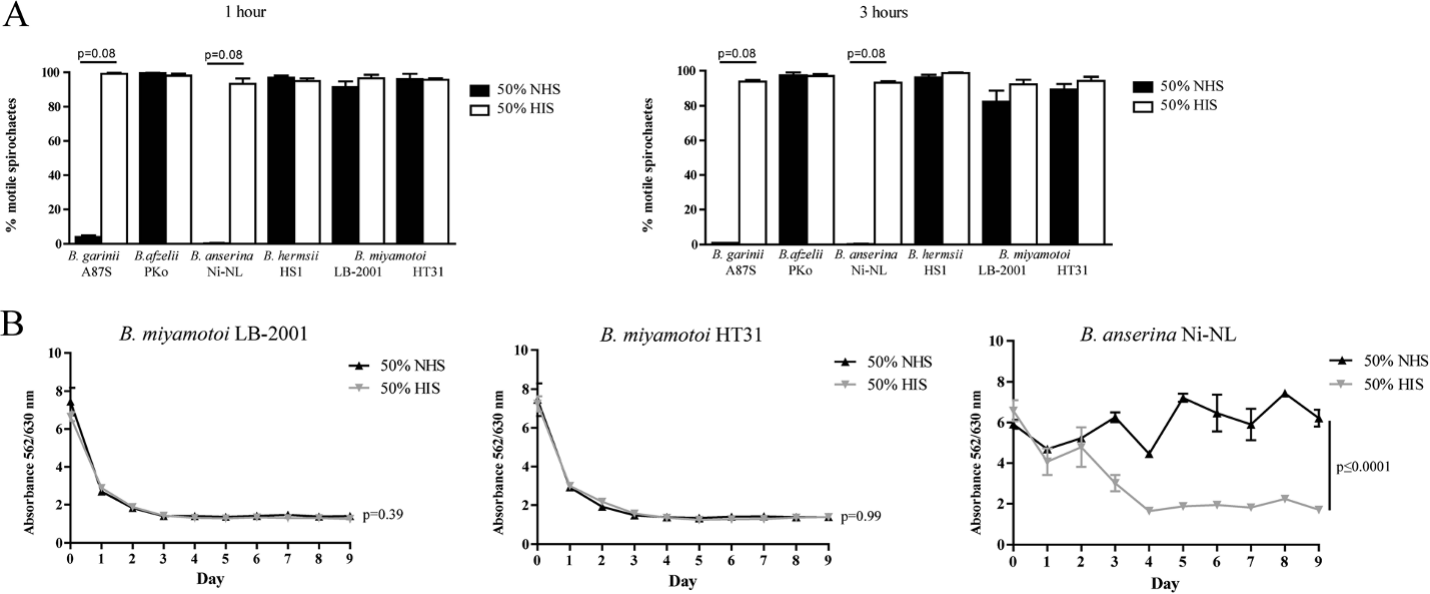
**Serum sensitivity: comparing motility and growth in normal human serum (NHS) versus heat-inactivated serum (HIS). A**. Direct killing assay. Six different *Borrelia* species were subjected to 50% pooled NHS or 50% pooled HIS: *B. afzelii* strain PKo, *B. garinii* strain A87S, *B. anserina* Ni-NL, *B. hermsii* HS1 and *B. miyamotoi* (LB-2001 and HT31). Blinded samples were examined by dark-field microscopy and 100 spirochetes per well were scored as either motile or immotile. Loss of motility in the NHS wells compared to HIS is indicative of complement mediated killing and inactivation of spirochetes. The figure depicts the mean, and error bars represent the standard error of the mean of triplicates from one representative experiment. A Kruskal-Wallis test was performed at t = 1 hour and t = 3 hours (p = 0.02 and 0.003, respectively) and for each strain motility between NHS and HIS incubation was compared using a two-tailed Mann–Whitney test (no significant differences). This experiment is representative of three different experiments. **B**. Growth inhibition assay. A total of 5x10^6^ spirochetes per well of *B. miyamotoi* LB-2001, *B. miyamotoi* HT31 or *B. anserina* Ni-NL were subjected to 50% NHS or HIS in the presence of a pH indicator (phenol red), cultivated at 33°C and absorbance at 562/630 nm was measured daily. A decrease in OD562/630 indicates decreasing pH due to spirochete growth. Error bars represent mean ± standard error of the mean (triplicates). This experiment is representative of two different experiments. The OD562/630 of spirochetes subjected to NHS versus HIS was compared using a repeated measures analysis of variance (ANOVA), and the p-value for interaction is depicted for each strain.

As humans have been infected by *B. miyamotoi*, we hypothesized the spirochete to be resistant to human complement. In order to evaluate serum sensitivity we grew *B. miyamotoi* LB-2001 and HT31 spirochetes to a concentration of 1-2x10^7^/ml and assessed spirochete motility one and three hours after addition of 50% pooled normal human serum (NHS). As a control, we added Heat Inactivated Serum (HIS), in which the complement was inactivated at 56°C. Indeed, at both time points there was no significant decrease in *B. miyamotoi* motility after addition of NHS as compared to HIS, indicating *B. miyamotoi* is resistant to human serum. As expected, *B. afzelii* PKo and *B. hermsii*, a Lyme borreliosis spirochete and relapsing fever spirochete respectively, were also resistant to killing by human complement (Figure 1A). In contrast, *B. garinii* A87S, a serum sensitive Lyme borreliosis spirochete, and *B. anserina*, a spirochete from the relapsing fever clade causing avian borreliosis, both showed a trend towards loss of motility after incubation with NHS compared to HIS (p = 0.08). We confirmed our findings using a pH-based growth inhibition assay (Figure 1B). In this pH-based assay growth of *B. miyamotoi* strains LB-2001 and HT31*,* represented by a pH-dependent decrease in OD, was similar when 50% normal human serum or 50% heat-inactivated human serum were added (p = 0.39 and p = 0.99, respectively). In contrast, *B. garinii* A87S and *B. anserina* did not grow in the presence of normal human serum while growth in heat-inactivated serum was unaffected (both p ≤ 0.0001, *B. garinii* data not shown). This clearly indicates that both *B. miyamotoi* strains are serum resistant, whereas *B. anserina* is serum sensitive. Negative controls (culture medium without spirochetes added) did not show a reduced OD over time (data not shown).

## Discussion and conclusions

In this study we describe a culture medium and method that can be easily used to culture *B. miyamotoi*. We were able to passage *B. miyamotoi* for more than 10 times under regular *Borrelia burgdorferi* culturing conditions, as well as in culture plates, in a modified Kelly-Pettenkofer medium with 10% added fetal calf serum (MKP-F). Independently of our efforts, other groups are developing alternative culture methods for *B. miyamotoi* (personal communication Volker Fingerle). Using our culture method, we discovered that *B. miyamotoi* is resistant to human serum. This means that *B. miyamotoi* can evade the human complement system, probably by using complement regulating surface proteins similar to other serum resistant *Borrelia* species. This evasion might partly explain the fact that humans can be infected with this spirochete, which seems to have adapted to humans as a host.

*B. hermsii*, another invasive relapsing fever spirochete, was first isolated by Kelly in 1971 [22] and his medium formed the basis for later Lyme borreliosis culture media. In 1982 Stoenner enriched this formulation, by adding CMRL (without glutamine) and yeastolate [23]. Barbour further adjusted the “fortified Kelly’s medium” to form BSK-I medium, using neopeptone as the peptone source and HEPES for buffering, while using CMRL 1066 with glutamine and omitting yeastolate [24]. In 1984 the medium was further improved to BSK-II medium by adding yeastolate and again omitting glutamine [25]. In 1986 researchers from the Max von Pettenkofer Institute altered the BSK media to culture *B. burgdorferi* sensu lato in what they called “modified Kelly medium”, and later referred to as “modified Kelly-Pettenkofer medium”, MKP medium [26]. Besides more subtle differences, MKP medium differs from BSK-I and BSK-II medium by the absence of glutamine and yeastolate, respectively. The similarity to these media is reflected by comparable isolation rates of *B. burgdorferi* sensu lato in MKP compared to BSK-II medium [27–29]. Because of previous observations that *B. miyamotoi* could not be serially passaged *in vitro* using BSK-II medium, in this study we cultured *B. miyamotoi* in MKP medium with the addition of 10% fetal calf serum, in an attempt to enhance growth of the pathogen. However, other formulations might also be suitable for culturing *B. miyamotoi*. Indeed, one might hypothesize that the addition of other serum types also results in successful cultivation, and we do not exclude the possibility that existing culture media can be adjusted to allow for *B. miyamotoi* cultivation without the need of additional serum. Regardless, in MKP-F, both strains showed robust replication in serial passages, and during the preparation of this manuscript we have been able to culture both strains for 15 passages without any loss in viability or peak densities (data not shown). Thus, using our formulation, we were able to culture two tick-derived *B. miyamotoi* isolates, but it still needs to be assessed whether our or other formulations are suitable for isolating the spirochete from clinical specimens, and what the exact role of fetal calf serum is in the *in vitro* propagation of *B. miyamotoi*. We did attempt to isolate *B. miyamotoi* from 350 μl of CSF from a patient who had a *B. miyamotoi* meningoencephalitis [9], however, this did not result in a positive culture, probably due the fact that the sample had been stored at −80°C for two years without the presence of glycerol. Culture efforts on fresh patient materials should be attempted in order to yield clinical isolates in the future.

Serum resistance is important in host invasiveness and reservoir host range for *Borrelia* spirochetes [11, 30]. *B. anserina*, *B. hermsii* and *B. miyamotoi* are phylogenetically related *Borrelia* species [31, 32]. We hypothesized that similar to *B. hermsii*, *B. miyamotoi* would be serum resistant, as these are both relapsing fever spirochetes able to infect humans, and *Borrelia anserina* to be serum sensitive. *B. anserina* is carried by *Argas* ticks which normally feed on birds and some species of which can cause anaphylactic reactions upon occasional human bites [33, 34]. Indeed, here we show that *B. miyamotoi* is serum resistant, whereas *B. anserina* is sensitive to human serum. *B. anserina* will probably have adapted to bird complement, as it is able to cause avian borreliosis [35], however, to our knowledge this remains to be investigated. Interestingly, a previous study showed that this spirochete was unable to bind human factor H, in contrast to *B. hermsii* [36]. This underscores the importance of factor H binding in serum resistance and host invasiveness. During the preparation of this manuscript another group has identified *B. miyamotoi* strain HT31 to be resistant to human complement, confirming the phenotype described in this paper [37]. More research is needed to identify the mechanism behind the complement resistance of *B. miyamotoi*, and we are currently investigating whether *B. miyamotoi* spirochetes express Complement Regulator Acquiring Surface Proteins (CRASPs).

Our culture method will further facilitate whole genome sequencing of *B. miyamotoi* strains including its plasmids as well as *in vitro* assays. In addition, the culture method described will be an impetus to basic and clinical research on this emerging human pathogen.

## Abbreviations

ANOVA: Analysis of variance
CRASP: Complement regulator acquiring surface protein
FCS: Fetal calf serum
GLPQ: Glycerophosphodiester phosphodiesterase
HIS: Heat-inactivated human serum
MKP: Modified Kelly-Pettenkofer medium
MKP-F: Variation on modified Kelly Pettenkofer medium with 10% fetal calf serum
NHS: Normal human serum
SCID: Severe combined immunodeficiency syndrome.
